# Charcot neuroarthropathy of the knee due to idiopathic sensory peripheral neuropathy

**DOI:** 10.1186/s12891-019-2873-9

**Published:** 2019-10-30

**Authors:** Qian-Hao Yang, Peichun Hsu, You-Shui Gao, Chang-Qing Zhang

**Affiliations:** 1 Department of Orthopedic Surgery, Shanghai Jiao Tong University Affiliated Sixth People’s Hospital, Shanghai, China; 2 Centre for Orthopaedic Research, Faculty of Health and Medical Sciences, The University of Western Australia, Nedlands, WA 6009 Australia

**Keywords:** Charcot arthropathy, Idiopathic sensory peripheral neuropathy, Knee

## Abstract

**Background:**

Charcot neuroarthropathy is a systemic disease that generates pathological changes in the musculoskeletal system, causing instability, dislocations, and deformities. Charcot neuroarthropathy of the knee, due to either diabetes mellitus or syringomyelia, is anecdotally reported with the epidemic of the diseases. However, idiopathic sensory peripheral neuropathy can inflict osteoarticular structures directly, inducing a dysfunctional Charcot neuroarthropathy. An early diagnosis and effective relief of the symptomatic deformity is essential for the treatment.

**Case presentation:**

We report the case of a patient with idiopathic sensory peripheral neuropathy who presented with a swelling right knee, as well as distorted and painless gait disorder, diagnosed as Charcot neuroarthropathy of the knee. Partial weight bearing with a hinged knee brace was used to correct the abnormal alignment and gait posture, and bisphosphonates were prescribed to decrease pathological bone resorption. Although the alignment and Knee Society Score got a gradual deterioration, the combination of orthosis and pharmacy could alleviate the symptom to a certain extent.

**Conclusion:**

The diagnosis of Charcot neuroarthropathy of the knee is rare that requiring early diagnosis. The presence of features, including painlessness, numbness, and deformed arthropathy following chronic-onset algesthesia loss should be taken carefully.

## Background

Charcot neuroarthropathy is a systemic disease that generates pathological changes in the musculoskeletal system, causing instability, dislocations, and deformities [1], first named by Jean Martin Charcot (1829–1893) in 1868 [2]. Diabetes mellitus and neurosyphilis are the most common causes of Charcot neuroarthropathy these days, mostly affecting the foot and ankle [3].

Charcot neuroarthropathy is reported to be closely associated with diabetes mellitus [4], syringomyelia [5], neurosyphilis [2], and idiopathic neuropathy [6], which might be misdiagnosed in many cases. With an increasing incidence of arthropathy, there is controversy about the treatment of Charcot neuroarthropathy in the knee. Total knee arthroplasty (TKA), arthrodesis, and conservative treatment should be considered appropriately in Charcot neuroarthropathy of diverse causes and stages. In the scenario of concomitant osteomyelitis, amputation might become the treatment of choice [7]. Herein, we present a case of Charcot neuroarthropathy of the knee following idiopathic sensory peripheral neuropathy.

## Case presentation

**Fig. 1:**
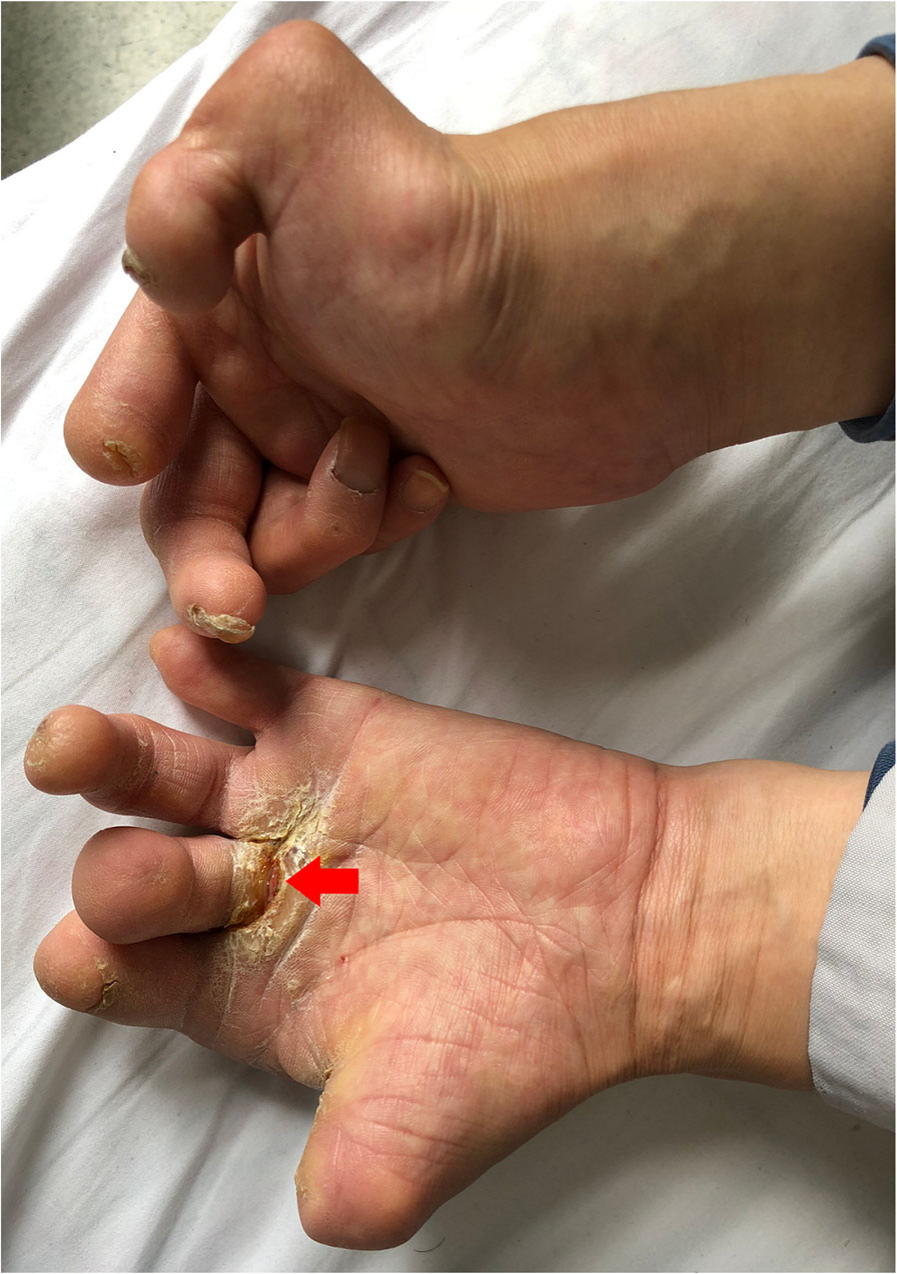
Multiple digital tips fall off naturally, in concomitant with uncontrollable and painless ulceration. The remnant fingers are deformed, with obvious palmar ulcer as indicated

A 40-year-old man presented to the orthopedic clinic with a 2-week history of swelling in the right knee and a 1-year history of painless gait disorder. He had previously been diagnosed with idiopathic sensory peripheral neuropathy, for which he had multiple fingertips falling off in his early teens and left leg amputation 5 years prior following massive painless ulceration (Fig. 1). The diagnosis of idiopathic sensory peripheral neuropathy follows the approach to the evaluation of peripheral neuropathies [8], evidenced as the unique clinical manifestation of distally predominant and symmetrical sensory abnormalities, and electrophysiological findings of mainly axonal damage. Charcot-Marie-Tooth disease was excluded due to his normal motor function. There was no history of diabetes mellitus and neurosyphilis. He also had no family history of inherited diseases.

**Fig. 2:**
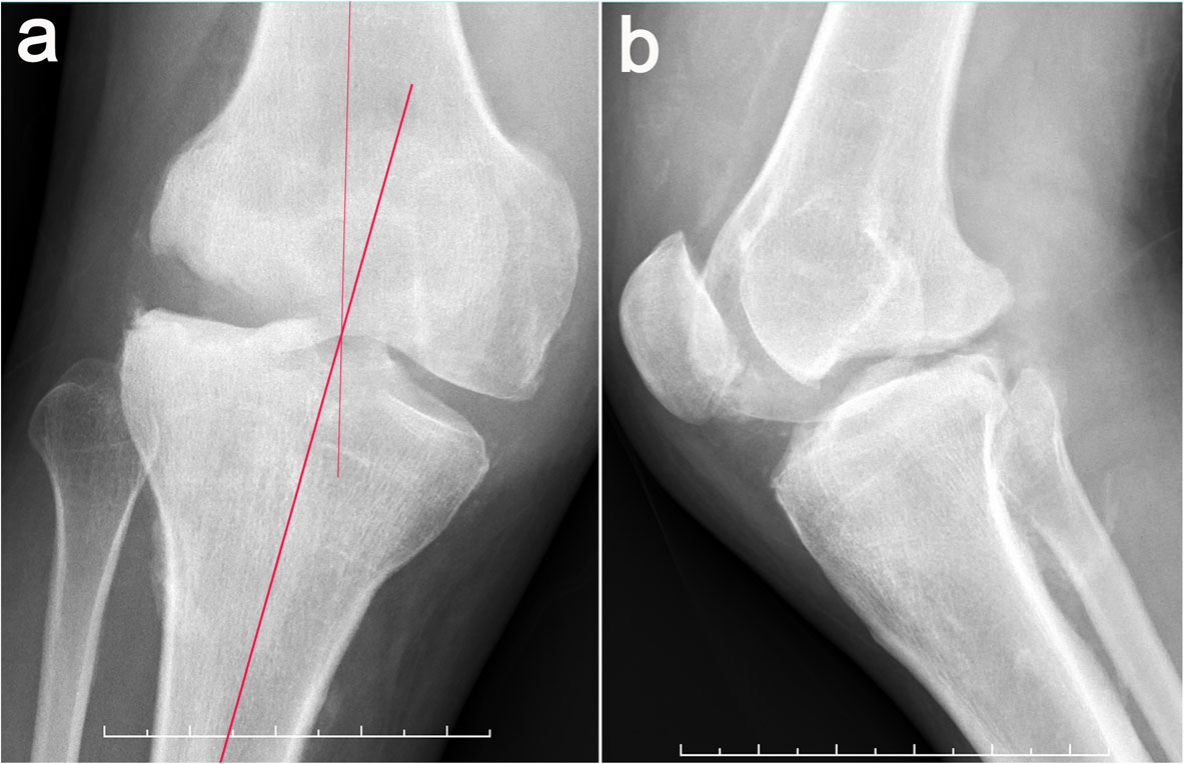
Anteroposterior (**a**) and lateral (**b**) plain radiographs of the right knee show a valgus knee, with a destructive femoral condyle. The knee is swollen, distorted, and painless, with a femorotibial angle of 165°

**Fig. 3:**
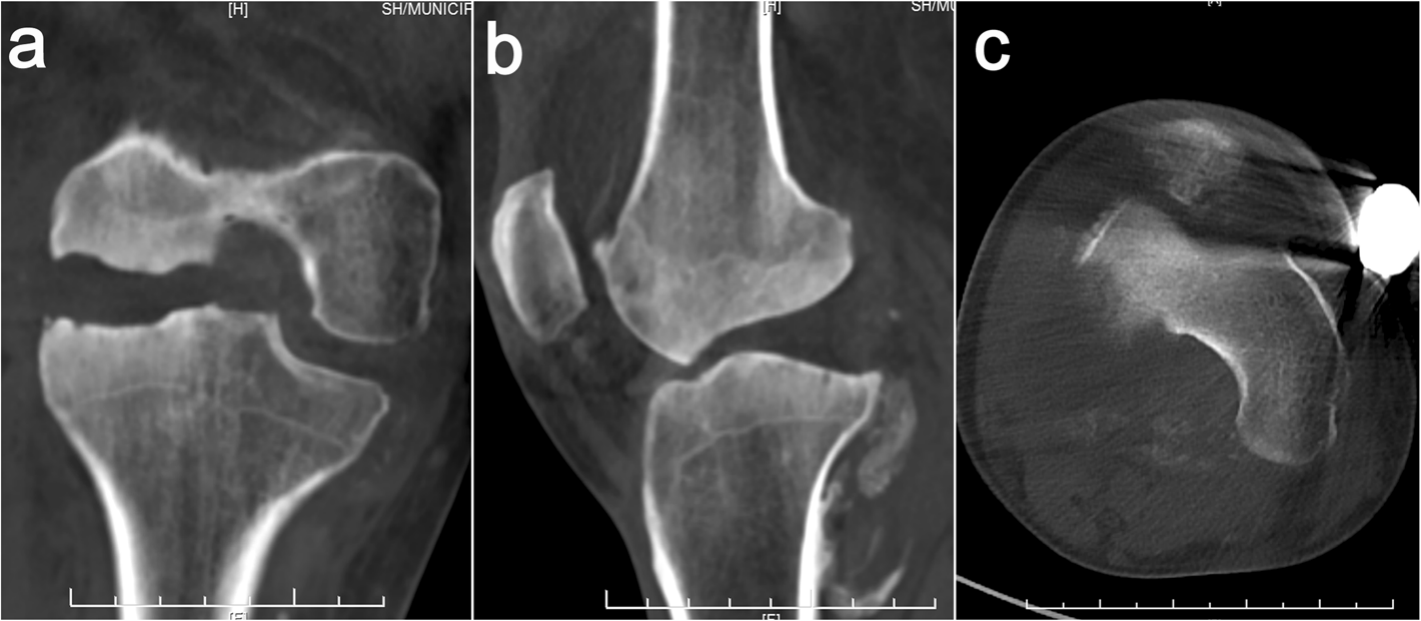
CT scanning of the right knee shows apparent widening of the joint space due to increased effusions (**a**) and aberrant morphology of lateral femoral condyle (**b**), with abnormal debris close to the posterior plateau (**b**, **c**)

**Fig. 4:**
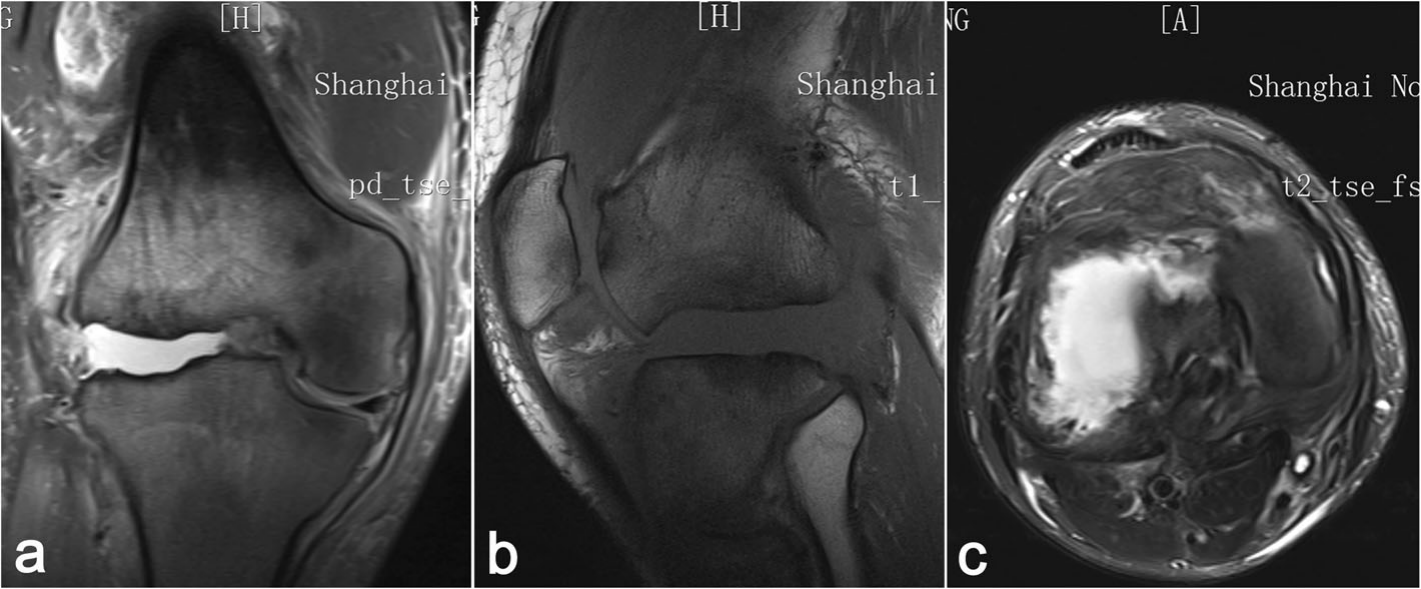
Magnetic resonance imaging reveals a wide disruption of anterior cruciate ligament, lateral collateral ligament and meniscus lateralis (**a**, **b**), with significantly increased effusions. The lateral femoral condyle is almost entirely destructed while the medial condyle is uninvolved (**c**)

On physical examination, a valgus distortion of the knee was noted, with significantly restricted ranges of flexion (105°) and extension (10°). Inspired by a recent study [9], we used the system of Knee Society Score (KSS) to evaluate the function. The knee score was 46 and the function score was 40 on admission. There was a slight deep tenderness over the superolateral aspect of the patella and the lateral femoral condyle. Despite gross deformity and distinct instability, he complained of no pain during daily activities and sleep. Paracentesis yielded bloody effusions, both negative for biochemical examinations and microbial culture. Laboratory studies showed slight elevation in alkaline phosphatase (ALP) levels (147 U/L; reference: 34-104 U/L) at admission, reflecting abnormal absorption of bone metabolism. Infection was excluded due to the normal ranges of leukocyte count (4.7 × 10^9^/L; reference: 3.5 × 10^9^–9.5 × 10^9^/L), C-reactive protein (10.36 mg/L; reference: 0.00–10.00 mg/L), and erythrocyte sedimentation rate (12 mm/h; reference: 0–21 mm/h) along with negative microbiological and cytological examination of the repeated fluid aspiration. The coagulation and syphilis tests were normal without evidence of hemophilic arthritis and syphilis-induced Charcot arthropathy. Plain radiographs and CT scans showed apparent widening of the joint space and multiple abrasions of the lateral femoral condyle, with abnormal debris close to the posterior plateau (Figs. 2, 3). Stress radiography was not conducted due to the high risk of pathologic fractures and incompetency of pain complaint. Plain radiographs, captured under supine and neutral position, showed the femorotibial angle (FTA) was 165°. Magnetic resonance imaging revealed a wide impairment of the anterior cruciate ligament, lateral collateral ligament, and meniscus lateralis with massive joint effusions (Fig. 4). Idiopathic sensory peripheral neuropathy-induced Charcot neuroarthropathy was diagnosed in collaboration with neurologists.

**Fig. 5:**
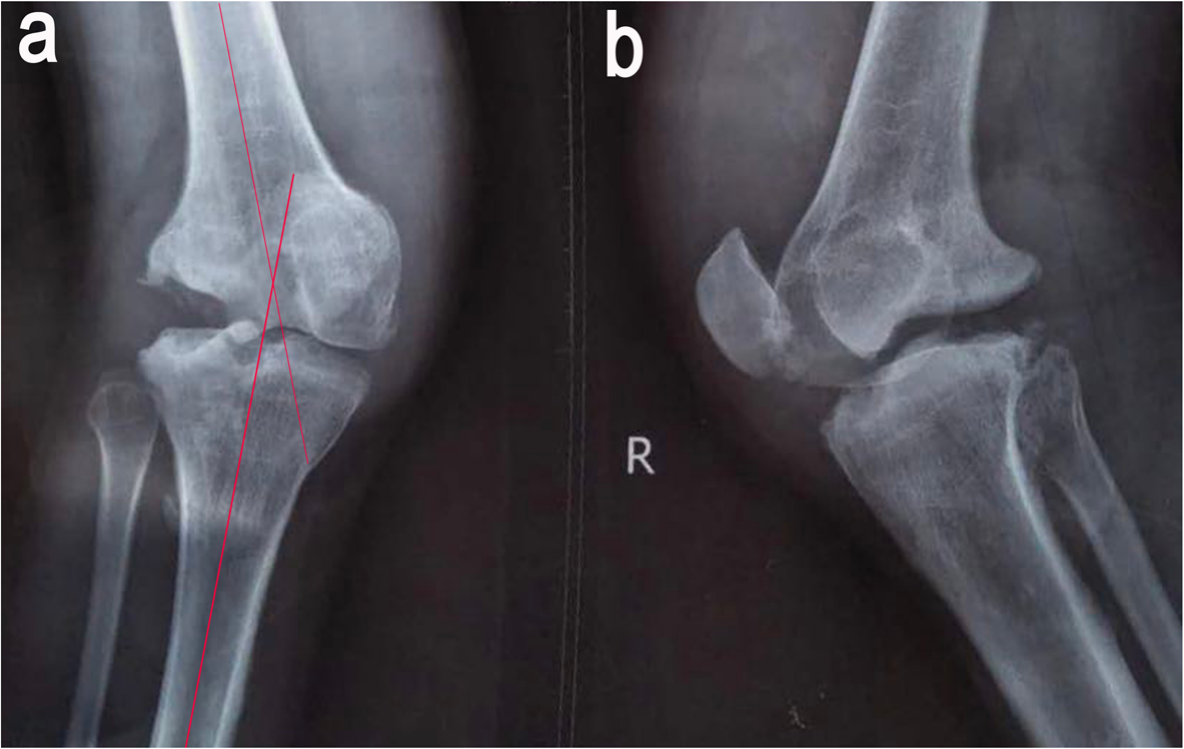
Anteroposterior (**a**) and lateral (**b**) plain radiographs of the knee at the 12-month follow-up showed the lesion was restricted in the lateral compartment. The femorotibial angle has progressed to 159°

Partial weight bearing with a hinged knee brace was advocated to correct the abnormal alignment and gait posture, followed by a gradual increase in flexion and extension over a 3-month period for clinical improvement. However, the significant disability determined the joint motion should be protected by the orthosis. Mild swelling of the knee was found in the lateral compartment, but there was no tenderness or redness. Additionally, he was administered physical therapy and rehabilitation along with bisphosphonates to decrease pathological bone resorption. ALP was dynamically monitored and found to be maintained between 108~134 U/L (reference: 50–136 U/L). Plain radiographs at the 12-month follow-up showed the valgus knee with the FTA of 159°, with a slight deterioration restricted in the lateral compartment (Fig. 5). The knee was reevaluated with KSS, showing the knee score dropped to 41 and the function score maintained 40. The range of motion was 10°~ 105°, reflecting the flexion and extension in the sagittal plane was not affected. Considering the potential risks of arthroplasty and inherent disadvantages of arthrodesis, protected weightbearing with the orthosis was recommended as the current treatment of choice [10].

## Discussion and conclusions

Historically, the 2 dominant pathophysiological causes of Charcot neuroarthropathy included the neurotraumatic (abnormal sensory innervation, repeated microtrauma, and unregulated inflammatory cascade) and neurovascular theories (hypervascular region in subchondral bone, vascular shunting, and subsequent osteopenia) [1, 3]. Peripheral neuropathy caused by diabetes mellitus is the main cause of Charcot neuroarthropathy of the knee. While the idiopathic sensory peripheral neuropathy in our case can cause chronic-onset loss of algesthesia, inducing hypalgesia, numbness, and deformed arthropathy. The primary degeneration of the dorsal root ganglion and trigeminal ganglion sensory neurons can cause gait ataxia, proprioceptive sensory loss, unnoticed damage, as well as repetitive microtrauma to the weight-bearing joint, contributing to Charcot neuroarthropathy [11, 12]. If not diagnosed and intervened promptly, it may result in severe complications, such as fractures and joint dislocations.

Charcot neuroarthropathy can occur secondary to syringomyelia, syphilis, diabetic neuropathy, alcoholic peripheral neuropathy, and idiopathic neuropathy. Diabetes is the most common etiology. Charcot neuroarthropathy is considered a major risk factor for below-the-knee amputation among the diabetics [1], while upper extremity can also be affected as a result of syringomyelia [5]. The diagnosis of Charcot neuroarthropathy is based primarily on thorough history and physical examination, including features, such as loss of protective sensation, presence of ulceration, and warm, swollen, erythematous foot and/or ankle. A combination of autonomic neuropathy, motor neuropathy, and sensory neuropathy results in osteopenia, joint instability, and increased loads on joints. The signs and symptoms of idiopathic sensory peripheral neuropathy-induced Charcot neuroarthropathy are consistent with non-length dependent nerve-fiber degeneration. The loss of sensory neurons in the dorsal root ganglia induce degeneration of short and long peripheral axons and central sensory projections in the posterior columns. Idiopathic sensory peripheral neuropathy induced Charcot neuroarthropathy, unlike others, is a rare disorder with unique characteristics, including deformities of fingertips and asymmetrical sensory disturbances.

Neuropathic arthropathy can be classified into four stages using standard radiology (prodromal, developmental, coalescence, and reconstructive) [10]. Identification of Charcot neuroarthropathy of the knee in Stage 0 and I have important therapeutic implications. The immobilization and non-weight bearing of the knee may prevent progression of skeletal destruction and deformity. Antiresorptive therapy, especially with bisphosphonates, has been revealed to have a modest effect on Charcot neuroarthropathy [13], inhibiting excessive osteoclast activation and proinflammatory cytokine response [14]. Total knee arthroplasty (TKA) and arthrodesis should be employed only in the reconstruction or coalescence stages [15], similar to a salvage operative treatment. Although TKA is satisfactory for most patients [16], the complications due to fragile bone and ligamentous laxity still exists [17], including periprosthetic fracture, aseptic loosening, instability, and infection [18]. Knee arthrodesis is accompanied by major functional limitations, resulting in the restriction of knee motion. Bisphosphonates were prescribed due to the nature of progressive destruction of Charcot neuroarthropathy. Bisphosphonates were beneficial to control the level of ALP, which was monitored through the observation. However, with a brief literature review, we found most of previous studies did not detect any biomarkers associated with bone absorption [5, 6, 9, 19–22]. Although Charcot neuroarthropathy is not a bone metabolism disorder in nature, a dynamic monitor of serum biomarkers can provide substantial reference to modulate bone homeostasis and alleviate skeletal destruction. The activity of osteoclast and underlying inflammation should be evidenced by essential mechanistic investigations in the future [1].

To the best of our knowledge, this is the first case of Charcot neuroarthropathy due to idiopathic sensory peripheral neuropathy. The patient had an impressive history of multiple fingertips abscission and left leg amputation following painless ulceration. Nerve conduction studies confirmed the presence of idiopathic sensory peripheral neuropathy. The swelling of the right knee and painless gait disorder were the primary clinical features, consistent with the diagnosis of neuropathic arthropathy of the knee. Moreover, only the lateral compartment of the knee was significantly involved, with an obvious valgus deformity, detected promptly before total joint destruction. Our case had satisfactory clinical outcomes from periods of nonoperative management in Stage I.

In summary, the diagnosis of Charcot neuroarthropathy of the knee is rare and is considered as the accumulated effect of peripheral neuropathy. Although an early diagnosis of Charcot arthropathy cannot alter natural course of the disease, it is beneficial to relieve symptoms and prevent severe complications. The presence of features, including painlessness, numbness, and deformed arthropathy following chronic-onset algesthesia loss increases the likelihood of idiopathic sensory peripheral neuropathy-induced Charcot neuroarthropathy.

## Data Availability

All data generated or analyzed during this study are included in this published article.

## Abbreviations

ALP: Alkaline phosphatase
FTA: Femorotibial angle
KSS: Knee Society Score
TKA: Total knee arthroplasty
